# Thin Film of Amorphous Zinc Hydroxide Semiconductor for Optical Devices with an Energy-Efficient Beneficial Coating by Metal Organic Decomposition Process

**DOI:** 10.1038/s41598-018-27953-6

**Published:** 2018-07-20

**Authors:** Makoto Karakawa, Tohru Sugahara, Yukiko Hirose, Katsuaki Suganuma, Yoshio Aso

**Affiliations:** 1 Institute for Frontier Science Initiative, Kanazawa University, Kakuma-machi, Kanazawa, Ishikawa, 920-1192 Japan; 2 The Institute of Scientific and Industrial Research, Osaka University, Mihogaoka 8-1, Ibaraki, Osaka, 567-0047 Japan

## Abstract

An effective metal oxide coating with solution processes by the metal organic decomposition method as deposited at room temperature (RT) poses great challenge. In this study, we report the characterization and evaluation of the semiconductor properties of a zinc hydroxide thin film with RT just as deposition by solution coating method. The films worked well as an inter-layer of the organic photovoltaic cell and optimized the film thickness condition with chemical and physical properties. As a result, we achieved a power conversion efficiency performance level, which was almost similar to that in the cells used after calcination in the crystal ZnO inter-layer. The presented process without any additional decomposition energy is expected to make a significant contribution to the realization of a flexible and cost-effective solution process for device fabrication.

## Introduction

Functional metal oxides have been investigated in a wide range and a variety of electronic device applications, such as transistors, memories, batteries, sensors, and superconductors. The properties of metal oxide semiconductors have bridged from semiconductor including organics to the metal of an electrical band structure. Hence, numerous research attempts have recently focused on organic electronics using metal oxides as inter-layers aligning the energy level between the organic semiconductor layer and the electrodes at the interface^1–8^. These researchers also realized various interesting functions when combined with organics. ZnO has recently been recognized as one of the candidates for the inter-layer, and such has become a hot topic^9^ for energy generation devices using n-Type (i.e., a hole-blocking and electron transporting layer of photo voltaic (PV) devices)^2,10^. Its good electron transporting properties caused by covalent binding displays a good wide-bandgap value of 3.4 eV and appropriate valence and conduction bands of −4.4 eV and −7.7 eV, respectively.

In making flexible, lightweight, and inexpensive organic electronic devices with a short pay-back time^11^, inter-layers must have low-cost coating during the device fabrication process. Many reports were released on the low-temperature calcination for the ZnO film fabrication, which clearly represented the demand for an annealing-free film fabrication. The standard solution process with zinc acetate requires an annealing step at over 300 °C^9,12^. Many research groups previously carefully investigated the effect of the annealing temperature and time on the ZnO properties in an attempt to decrease the process temperature of 80 °C to 150 °C^13,14^. Decreasing the annealing temperature inevitably leads to the formation of an amorphously structured ZnO and/or mixing Zn-related hydroxide (ZnOH_x_) with insufficient electric properties. L.K. Jagadamma *et al*. recently demonstrated the good performance of amorphous ZnO fabricated at 100–110 °C^15^ as an organic photo voltaic (OPV) cell inter-layer, suggesting the possibility of using amorphous films in electric devices.

We demonstrate herein that room temperature (RT)-processed ZnO_x_ and ZnOH_x_ films prepared through the metal organic decomposition (MOD) method can be successfully used as the inter-layer of OPV cells. The X-ray photoelectron spectroscopy (XPS) and ultraviolet (UV) spectroscopy analyses revealed a mixing oxide and hydroxide binding state of ZnOH_x_ with an amorphous structure, which resulted in an amorphous zinc hydroxide [a-ZnOH_x_] film. As reported in our previous literature, the a-ZnOH_x_ film obtained from the precursor could be converted into a completely crystallized ZnO (c-ZnO) after calcination at 300 °C for 5 min^12^. Several thicknesses of the a-ZnOH_x_ inter-layers were evaluated with the diode characteristics in OPV cells for comparison with c-ZnO with conventional solution processes. OPV cells, including the a-ZnOH_x_ inter-layer, showed the same order of OPV performance to that of a cell containing the c-ZnO layer. This result implied that a-ZnOH_x_ films can be adapted for use as the inter-layer of OPV cells as long as thin films are used as the semiconductors.

## Result and Discussion

The precursor solution was prepared by mixing ZnAc, 2-methoxyethanol, and ethanolamine at 65 °C overnight and avoiding any humidity until the solution became transparent. The precursor concentration was 100 mM/L. The prepared precursor was diluted to 50, 30, 20, and 10 mM/L with ethanol, then spin-coated on indium tin oxide (ITO) to create the inter-layer. An OPV inter-layer sample prepared using 100 mM/L precursor was calcined at 300 °C for 5 min for comparison. The films were dried for 10 min in inert atmosphere after spin-coating. The organic layer (PTB7:PC_71_BM, 1:1.5 in chlorobenzene with 3% 1,8-diiodooctane) was then spin-coated on top of the dried film without any annealing step.

**Figure 1:**
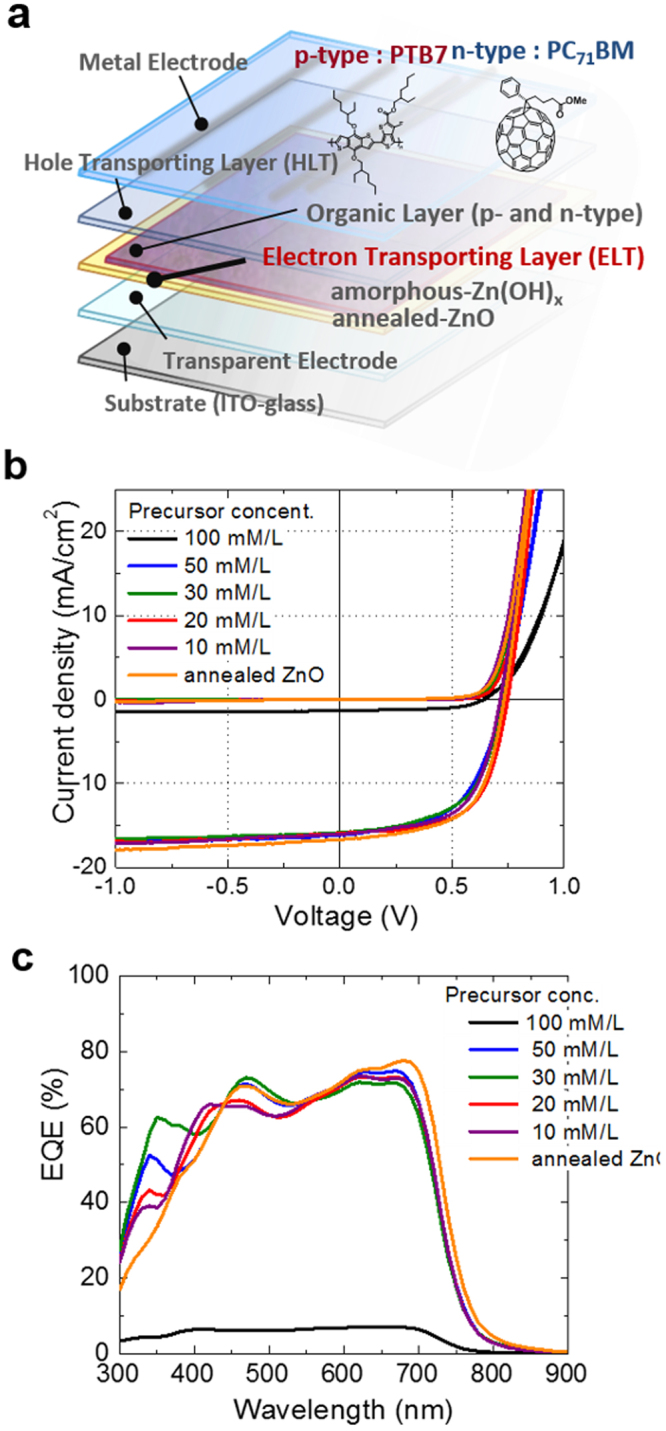
(**a**) Structure of the OPV cells using the ZnOH_x_ and ZnO inter-layers in this study. The performance (**b**) linear plots and (**c**) external quantum efficiency of the cells.

After OPV cell fabrication (schematic, Fig. 1a), the current density–voltage (*J*–*V*) curves and external quantum efficiencies (EQE) of the cells were measured under dark conditions and illumination with light (AM 1.5) at 1000 W/m^2^ as seen in Fig. 1b,c. Table 1 summarizes the OPV performance data with the thickness of the inter-layers and the diode data from the dark conditions. The average power conversion efficiency (PCE) data from 10 devices were also calculated.

**Table 1:** Output parameters of organic photovoltaic cells using ZnOH_x_ inter-layer.

Precursor conc.	100 mM/L^b^	50 mM/L^b^	30 mM/L^b^	20 mM/L^b^	10 mM/L^b^	ZnO^c^
Thickness [nm]	60 ± 5	40 ± 3	20 ± 3	10 ± 3	<5	ca. 35 ± 5
*J*_*sc*_ [mA/cm^2^]	1.348	16.153	15.836	15.950	16.020	16.693
*V*_*oc*_ [V]	0.645	0.728	0.725	0.748	0.716	0.733
FF	0.561	0.555	0.572	0.624	0.603	0.608
PCE^a^ [%]	0.49(0.47)	6.52(6.04)	6.57(6.06)	7.44(7.25)	6.91(6.34)	7.44(7.26)
Forward voltage [V]	0.57	0.55	0.55	0.59	0.51	0.47

^a^averaged values on 10 devices in parentheses, ^b^concentration of precursors, ^c^annealed at 300 °C for 5 min.

Figure S1 shows the effect of the inter-layer thickness on the dark *J*–*V* characteristics. The rectification ratio of the OPV devices of a-ZnOH_x_ inter-layer was very high around 10^4^ at the region from −1 to 1. Moreover, the values decreased down to 10^2^ when the inter-layer thickness was decreased. However, the values with the conditions of the RT coating had higher levels compared with the c-ZnO inter-layer (Figs 1b and S1a,b). The leak currents of the reverse bias voltage region decreased with an increase in the thickness. Conversely, the forward voltages increased at the forward bias voltage (Fig. S1c–d and Table 1). These electrical properties of the OPV devices were on the theoretical representation of the diodes. The shunt and series resistance decreased when the leak current decreases. However, these changes in resistance were in a trade-off relationship with the OPV characteristics, especially the fill factors. Therefore, finding the optimum value of the film thickness was necessary in this study.

All the OPV cells exhibited good FF values despite the annealing-free inter-layer in the illumination condition, indicating that the RT-processed film was active in the electrical cells without the need for an annealing step. The OPV cell containing the film from the 100 mM/L precursor showed very a low short-circuit current density (*J*_sc_). In contrast, the OPV cells with the inter-layers derived from the diluted precursor series showed a satisfactory performance and especially showed good *J*_sc_ values. The *J*_sc_ and *V*_oc_ values were surprisingly improved by the precursor dilution to 50 mM/L from 100 mM/L. Moreover, the 20 mM/L precursor afforded the highest PCE, which was comparable to that of the cell with the calcined ZnO inter-layer. The cell derived from the precursor diluted to 10 mM/L and higher exhibited a decreased OPV cell performance because of a decreased *V*_oc_. However, further dilution confirmed the decreasing *V*_oc_ trend (Table S1). *V*_oc_ exhibited a similar behavior to that seen for the bare ITO upon over-dilution (i.e., without a cell inter-layer), but the *J*–*V* curves were completely different with those of the bare ITO. In the case of the over-dilution precursor, some ultra-thin parts in the inter-layer were covered on the ITO surface, and preventing the occurrence of a leak current was difficult. The band of a-ZnOH_x_ semiconductor at interfaces ITO might be pulled up by the Schottky barrier junction almost the same level of ITO work function (Fig. S2), when the semiconductor inter-layer was ultra-thin (e.g., approximately less than 3–5 nm in this case). The hole and electron barrier effect might be decreased resulting increase an opportunity for charge recombination in the case of over diluting condition leading ultra-thin film such as 10 mM/L. As a consequence, the optimized thickness of the a-Zn(OH)x films were approximately 20 nm.

Transmittance mode UV–vis absorption measurements of the films were performed on a quartz substrate to clarify the reasons for the good functionality of the inter-layer fabricated from the diluted precursor and the RT process. The spectra (Supplementary Fig. S3) indicated that the films had a high transmittance in the visible light region and absorbed UV light. The calculated optical gaps of the films were over 4.4 eV. The absorption observed from 500 nm to 700 nm might be assigned to scattering by nano-sized effect of metal oxides. Considering the fact that calcined ZnO absorbs UV light from 300 nm and below, X-ray photoelectron spectroscopy is a useful and powerful analytical method of determining the composition of materials from the binding energy of their constituent atoms.

**Figure 2:**
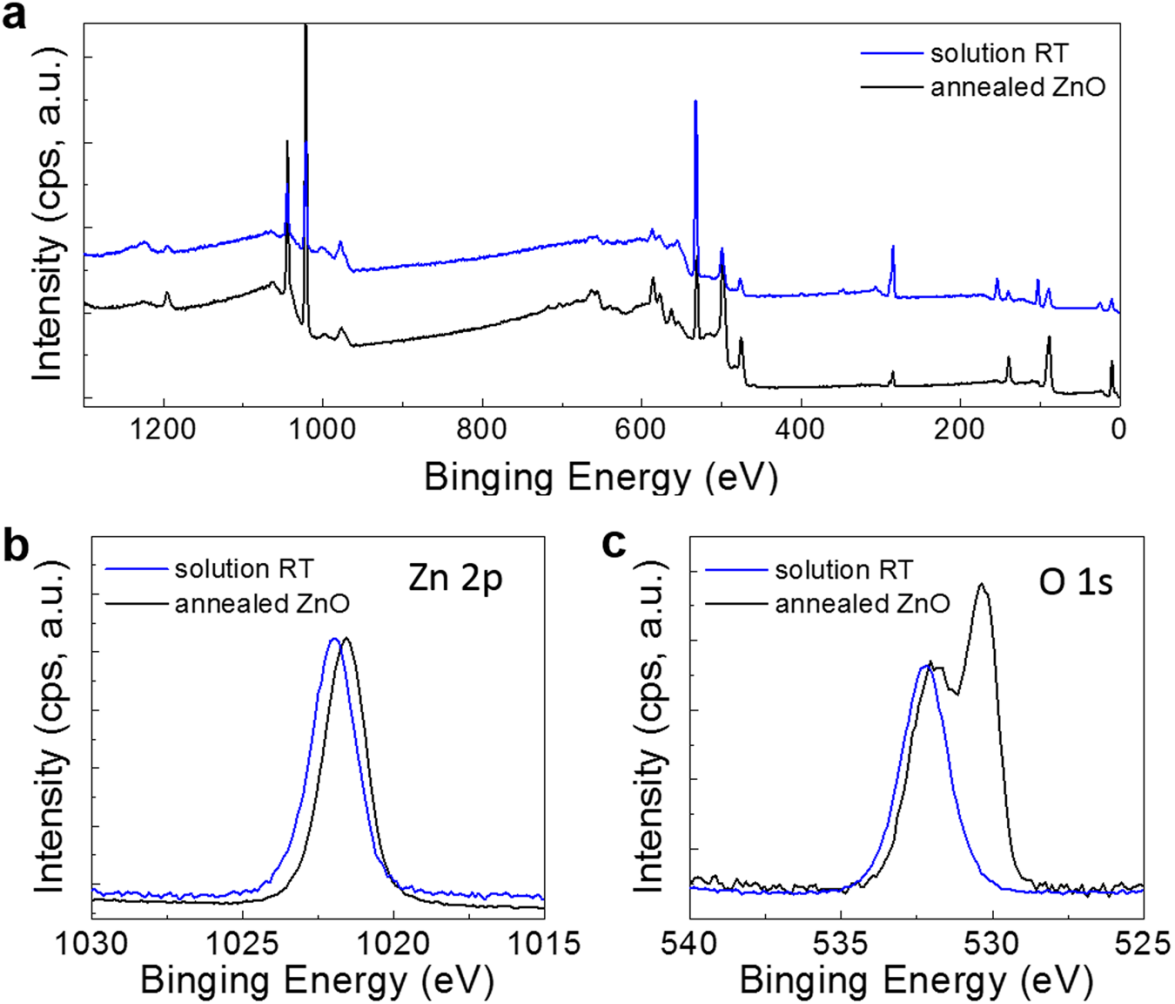
XPS analysis of Zn-related thin-films. (**a**) XPS survey spectra and local spectra of (**b**) Zn 2p, and (**c**) O 1s of ZnOH_x_ thin films and annealed ZnO.

Figure 2 shows the obtained survey and narrow spectra of the binding energies of zinc (Zn 2p: Fig. 2b), oxygen (O 1s: Fig. 2c), nitrogen (N 1s: Fig. S4a), and carbon (C 1s: Fig. S4b) for the solution RT (20 mM/L) and annealed films. The survey spectrum for solution RT is same as reported data^16^. The Zn-binding energy peak of the solution RT films was shifted compared with that of the annealed-ZnO (Fig. 2b). The coordination state of the ligands around the Zn atoms was slightly different in both films, which was attributed to their oxides or hydroxides OH. The main peak of the solution RT film in the oxygen spectra in Fig. 2c appeared at 532.2 eV, which could be assigned to the hydroxyl groups attached to Zn atoms (metal-OH bonds). Meanwhile, the annealed-ZnO film was composed of a mixture of both ZnO and ZnOH_x_ because of the M-OH and M-OC bonds observed at 532 eV, and M-O-M bonds observed at 530 eV of the thin film. These results indicated that ZnOH_x_ contributed to the device properties of the c-ZnO film, implying that the metal hydroxide may have acted as a semiconductor in the thin film state in the buffer layer. Our results might break the fixed idea that crystalline oxide films are indispensable for electronic device applications under the use of an ultra-thin film, such as the inter-layer of an OPV cell used here. The observed Zn 2p and O 1 s narrow peaks may support the possibility that the ZnOH_x_ in the calcined ZnO might contribute to its performance in the OPV device, as discussed earlier.

The N content of the film (Fig. S4a) originated from the 1-amino-ethanol used to form a chelate structure in the precursor^17^. The conditions used to produce c-ZnO (300 °C annealing) were sufficient to cause the N component to vaporize. Therefore, a very low or undetectable content of N in the c-ZnO film was reasonable. Surprisingly and fortunately, the N content of the annealing-free film from the diluted precursor was also very low although 1-amino-ethanol has a high boiling point of 145 °C.

The C atoms likely originated from both the precursor reagent and the contamination from the ambient air before the XPS measurements (Fig. S4b). The same order of C content was observed in all samples, and was sufficiently low in the annealing-free films that the film properties for the device used were expected to be unaffected.

**Figure 3:**
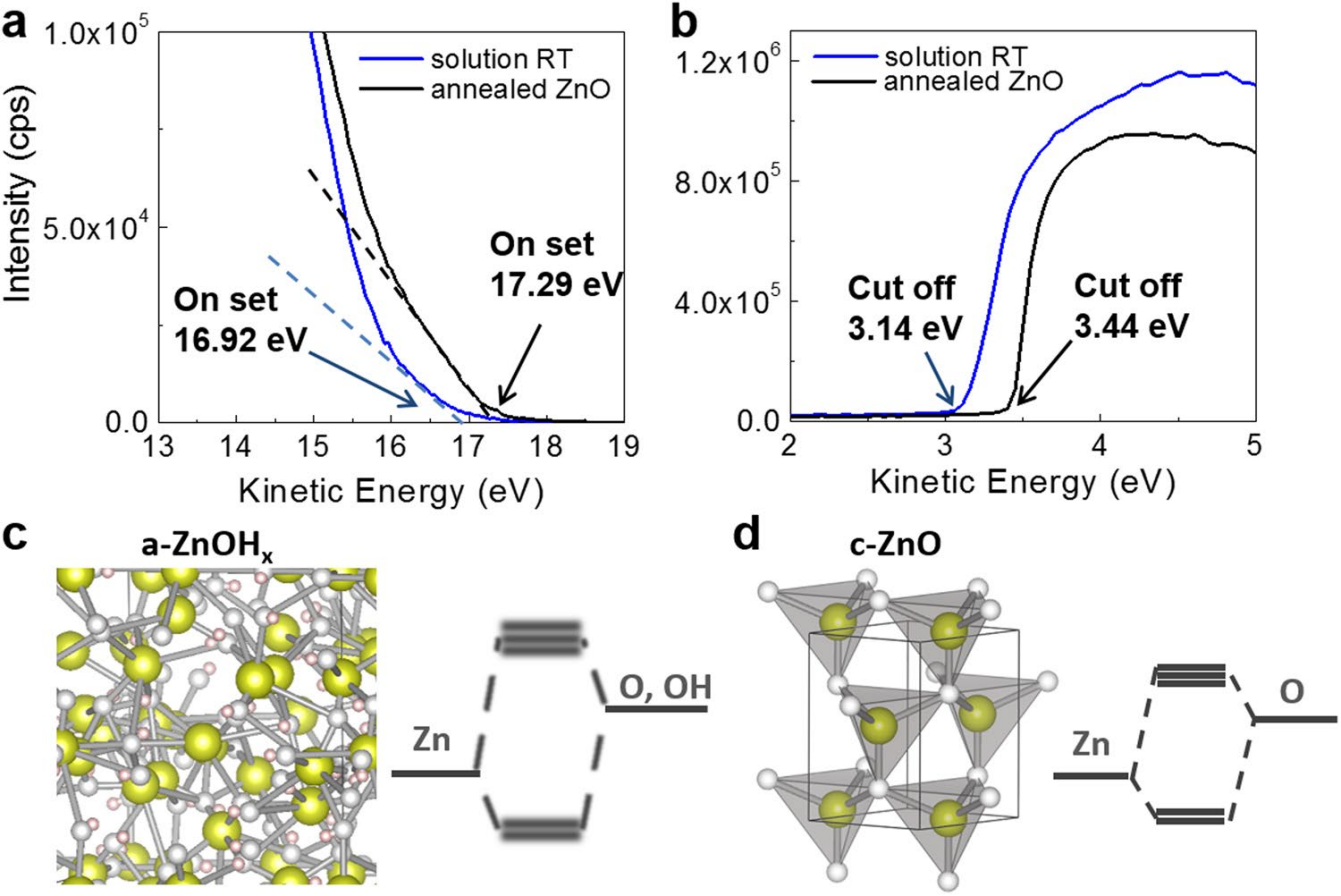
UPS spectra of room temperature processed solution RT (20 mM/L) and annealed ZnO films. (**a**) On set and (**b**) Cut off regions. Schematic images and bind structures for (**c**) a-ZnOH_x_ and (**d**) c-ZnO.

The XPS analysis results clearly showed that the annealing-free films fabricated using the RT solution process were composed of ZnOH_x_, and may uncrystallize the distributed structure: an amorphous ZnOH_x_ [a-ZnOH_x_]. Ultra-violet photoelectron spectra were collected from the thin films of a-ZnOH_x_ on ITO glass substrates to study the energy levels of the a-ZnOH_x_ film. Figure 3 shows the spectra of a-ZnOH_x_ and c-ZnO around the kinetic energy of on-set and cut-off. The band tail on the valence band maximum (VBM) level may have been derived from the amorphous structure of the a-ZnOH_x_ (Fig. 3a). The broad band structure (band fluctuation) of the annealing-free film at the VBM arose from unstable charge transfer states. The disordering of the degenerate electron orbital overlapping on the crystalline field was split through the randomly distributed metal cations and anions in the amorphous state (Fig. 3c). However, the VBM level of a-ZnOH_x_ was increased by its amorphous state, thereby resulting in an on-set value of 16.92 eV, which was similar to the value of 17.29 eV measured for c-ZnO (Fig. 3d).

The thin films of a-ZnOH_x_ had a slightly shallower work function (WF, −3.14 eV) and a lower-lying valence band (VB, −7.44 eV) compared to those of c-ZnO (i.e., WF: −3.44 eV and VB: −7.36 eV). The conduction band level of a-ZnOH_x_ (−3.04 eV) was estimated using the optical band gap (4.4 eV) measured from its UV–vis absorption spectrum. These measured energy levels were favorable for the application of a-ZnOH_x_ as an OPV cell inter-layer.

**Figure 4:**
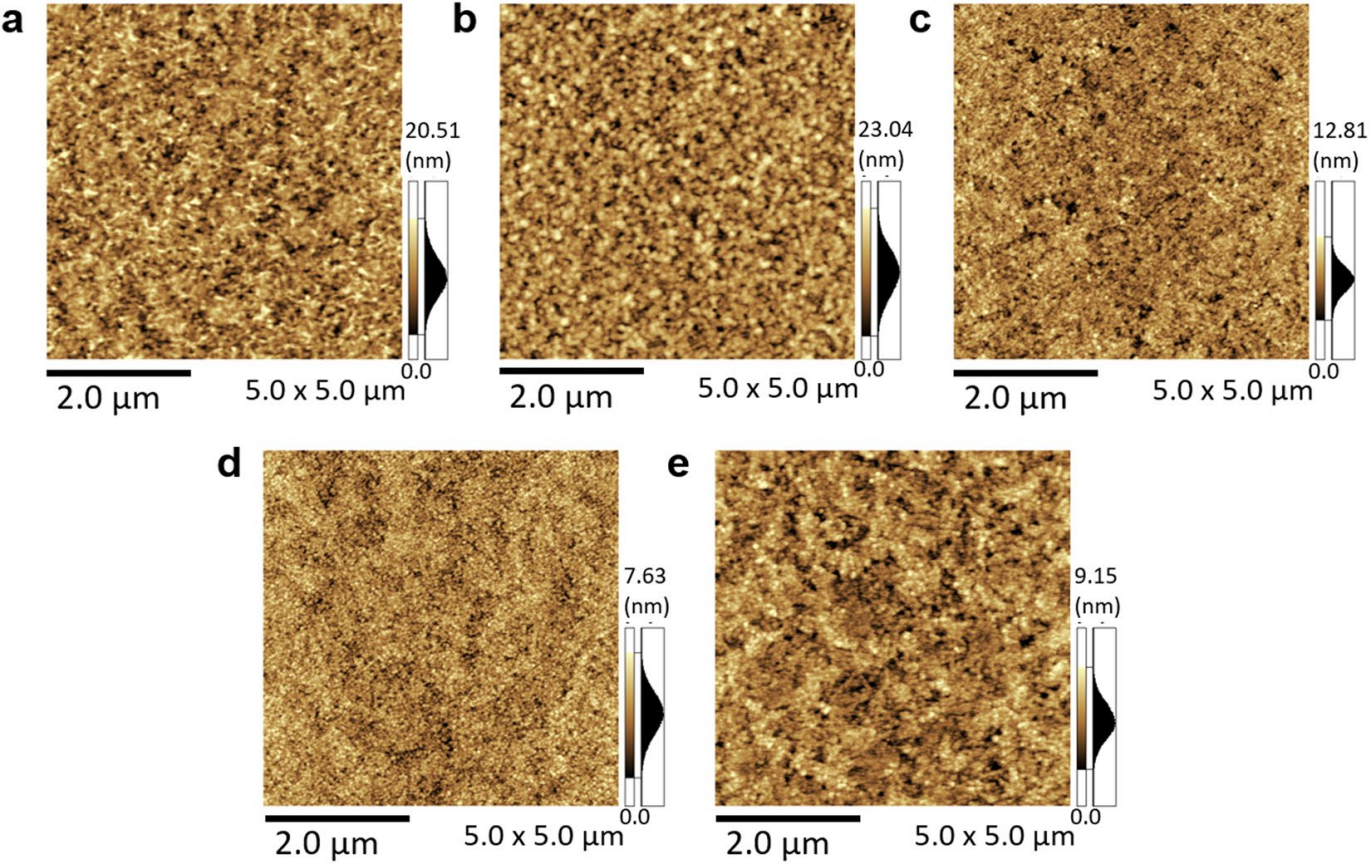
Surface morphology of films fabricated with different concentrations of ZnOH_x_ precursor. (**a**) 100 mM/L, (**b**) 50 mM/L, (**c**) 30 mM/L, (**d**) 20 mM/L, and (**e**) 10 mM/L.

As described earlier, the UV–vis and XPS measurements revealed that the films were mainly composed of a-ZnOH_x_. Accordingly, atomic force microscopic (AFM) analyses were conducted using real device surfaces to clearly study the relationship between the film fabrication conditions and the OPV properties (Figs 4 and S5).

The OPV cell containing an inter-layer fabricated with the 20 mM/L precursor provided the highest PCE of 7.4% (an average over 7% in Table 1). An AFM surface image of the inter-layer (20 mM/L precursor) revealed that it was constructed of closely-aggregated nano-ordered grain-like particles. In the case of the 10 mM/L precursor, some ultra-thin parts in the inter-layer were covered on the ITO surface, and preventing the leak current occurrence was difficult. The conduction band minimum (CBM) level of the ultra-thin (i.e., a few nano-meters) inter-layer pulled up the ITO work function (metal side) level similar to a Schottky junction.

The reproducibility experiments indicated that the nano-ordered grain-like structures controlled by the precursor concentration and preparation conditions were indispensable for favorable electrical properties. Figure S5 depicts example AFM images of the samples that exhibited a poor OPV performance. These films had relatively larger-sized grains than those shown in Fig. 4. These results confirmed that the aggregated grain size was an important factor in obtaining a good inter-layer for good OPV cells. All poor devices were fabricated with a large-grained a-ZnOH_x_ inter-layer.

## Conclusion

This study reported the potentials of thin films fabricated by a RT solution process as OPV inter-layers. It is a new discovery that semiconductor characteristics are revealed by the film. The thin films obtained from the precursor concentrations tuned with ethanol dilution, and were analyzed by UV–vis, XPS, UPS, and AFM measurements. The films produced using the present solution process were mainly composed of ZnOH_x_. This ZnOH_x_ was discovered to have excellent electrical properties. The a-ZnOH_x_ ultra-thin films exhibited a significantly good performance as an OPV cell inter-layer compared to that of the conventional c-ZnO film. Considering the procedures used to fabricate the thin films, the a-ZnOH_x_ film has distinct advantages over the c-ZnO film. The a-ZnOH_x_ inter-layer is a promising material for OPV cells because these cells must be fabricated with low-cost roll-to-roll printing processes. We believe that the amorphous ZnOH_x_ film will be recognized as candidate for future devices such as flexible devices.

## Experimental Details

### Materials

The reagents were purchased from Wako Pure Chemical Industries, Tokyo Kasei Chemical Industries, and Sigma Aldrich and used without further purification. PTB7 for the OPV cells was purchased from 1-Material Chemscitech and used as received.

### Measurements

The UV–vis spectra of the a-ZnOH_x_ thin-films on quartz plates (99% transmittance at 550 nm) were recorded with a Shimadzu UV-3100PC. The surface morphologies of the metal oxide films on the ITO glass were observed using an atomic force microscope (Shimadzu, SPM9600). X-ray photoelectron emission spectroscopy and ultraviolet photoelectron spectroscopy were performed using a Shimadzu-Kratos AXIMA-NOVA that utilized 300 W Mg Kα radiation with 0.1 eV steps and operated at a base pressure of 10^−8^ Pa. UPS was conducted under high vacuum (10^−8^ Pa) with a monochromatic He ultraviolet source He I (*hυ* = 21.22 eV). The FT-IR ATR spectra were recorded using a Perkin Elmer FT-IR Frontier with an ATR attachment. The thicknesses of the deposited solution RT films were measured using a KLA-Tencor alpha-step IQ surface measurement profiler.

Solution precursor preparation procedure: The ZnO precursor was prepared by metal organic decomposition according to the literature^8^. To guaranteed quality of precursor, fresh Zinc acetate dihydrate should be used and care a humidity of atmosphere. Zinc acetate dihydrate [Zn(CH_3_COO)_2_∙2H_2_O] (92 mg, 0.5 mmol) from Wako Pure Chemical Industries, Ltd. was dissolved in 2-methoxyethanol (5 mL) with monoethanolamine (30 mg, 0.5 mmol) as a stabilizer. The total molar concentration of the precursor was controlled to 0.1 M/L and stirred (ca. 300–400 rpm) at approximately 65 °C using a hot water bath for 6–12 h until a transparent homogeneous sol solution was obtained. Prior to spin coating to form the inter layers for the OPV devices, the sol-solution was diluted to 50, 30, 20 and 10 mM/L using absolute ethanol in glove box an inert atmosphere at 20–25 °C.

OPV device fabrication and measurements: Glass slides patterned with ITO (89% transmittance at 550 nm, Sanyo Vacuum Industries Co., Ltd. Japan) were treated in an O_2_ plasma oven for about 5 min. The cleaned ITO glass substrates were spin-coated (1000 rpm for 60 s) with the solution RT precursor (100, 50, 30, 20 and 10 mM/L) and dried in an inert atmosphere. The organic active layer was prepared by spin-coating a blended solution of PTB7:PC_71_BM (1:1.5, wt/wt, concentration of 25 mg/mL) in chlorobenzene, including 1,8-diiodooctane (3%) at 1000 rpm for 2 min. A 10 nm MoOx layer and a 100 nm Ag layer were subsequently evaporated through a shadow mask to define the active area of the devices (3 × 3 mm) and form a top anode. The solar cells were subsequently tested under 1.5 G simulated air mass (AM) solar irradiation (1000 W/m2, XES-301S, San-Ei Electric Co., Ltd. Japan). The current density–voltage (*J–V*) characteristics of the films were recorded using a PC-controlled Keithley 2400 source meter.

## Electronic supplementary material

Supplementary Information
