# Supplementary material for: Thin Film of Amorphous Zinc Hydroxide Semiconductor for Optical Devices with an Energy-Efficient Beneficial Coating by Metal Organic Decomposition Process
Source: Sci Rep. 2018 Jul 20;8:10839. doi: 10.1038/s41598-018-27953-6 (PMC6054676; doi:10.1038/s41598-018-27953-6)
Supplement: Supplementary file 1 — Supplementary Information [file 41598_2018_27953_MOESM1_ESM.doc]

Supporting Information

Thin Film of Amorphous Zinc Hydroxide Semiconductor for Optical Devices with an Energy-Efficient Beneficial Coating by Metal Organic Decomposition Process

Makoto Karakawa*, Tohru Sugahara*, Yukiko Hirose, Katsuaki Suganuma, Yoshio Aso

**Table S1.** Output parameters of organic photovoltaic cells using ZnOH_x_ buffer layer using different precursor concentrations

| Precursor conc. | *J_sc_* [mA/cm^2^] | *V_oc_* [V] | FF | PCE^a^ [%] |
| --- | --- | --- | --- | --- |
| rt-ZnOH 100 mM^b^ | 1.279 | 0.658 | 0.566 | 0.47 (0.49) |
| rt-ZnOH 50 mM^b^ | 15.467 | 0.720 | 0.543 | 6.04 (6.52) |
| rt-ZnOH 30 mM^b^ | 15.126 | 0.713 | 0.562 | 6.06 (6.57) |
| rt-ZnOH 20 mM^b^ | 16.139 | 0.732 | 0.613 | 7.25 (7.44) |
| rt-ZnOH 10 mM^b^ | 16.131 | 0.704 | 0.558 | 6.34 (6.91) |
| rt-ZnOH 5 mM^b^ | 15.955 | 0.691 | 0.573 | 6.33 (6.57) |
| rt-ZnOH 2.5 mM^b^ | 15.743 | 0.672 | 0.538 | 5.70 (6.20) |

^a^ the highest values in parentheses, ^b^ concentration of precursors


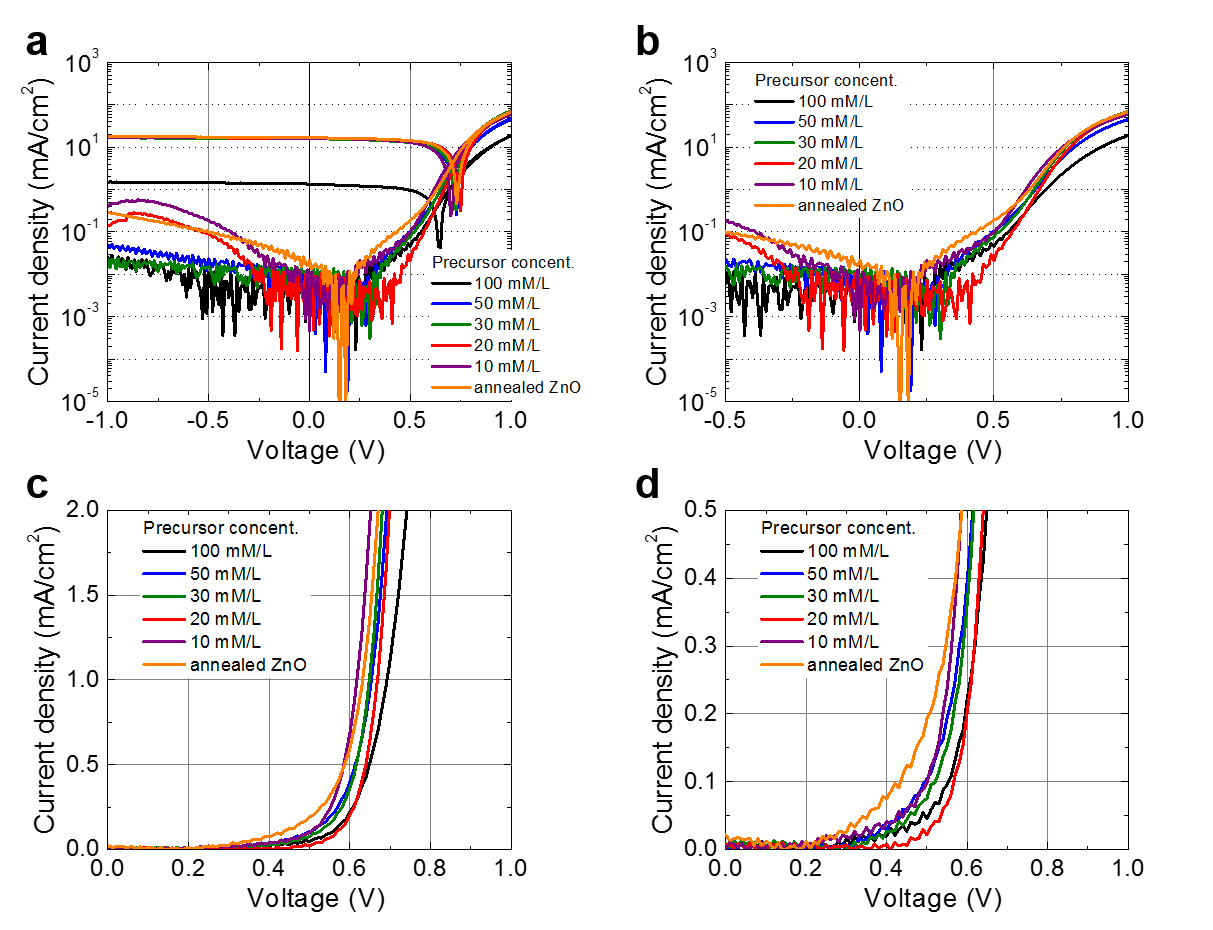


**Figure S1.** Current density-voltage plots curves of the OPV cells performance using the ZnOH_x_ inter layers. Logarithmic scales of (a) current density vs full scale voltage of illumination and dark condtions, and (b) the dark condtion of current density-voltage curves　from -0.5 V to 1.0 V, (c) the diode characteristics (in dark condtion) as a region of forward voltage and (d) expanded Y-axis from 0 to 0.5 mA/cm^2^.


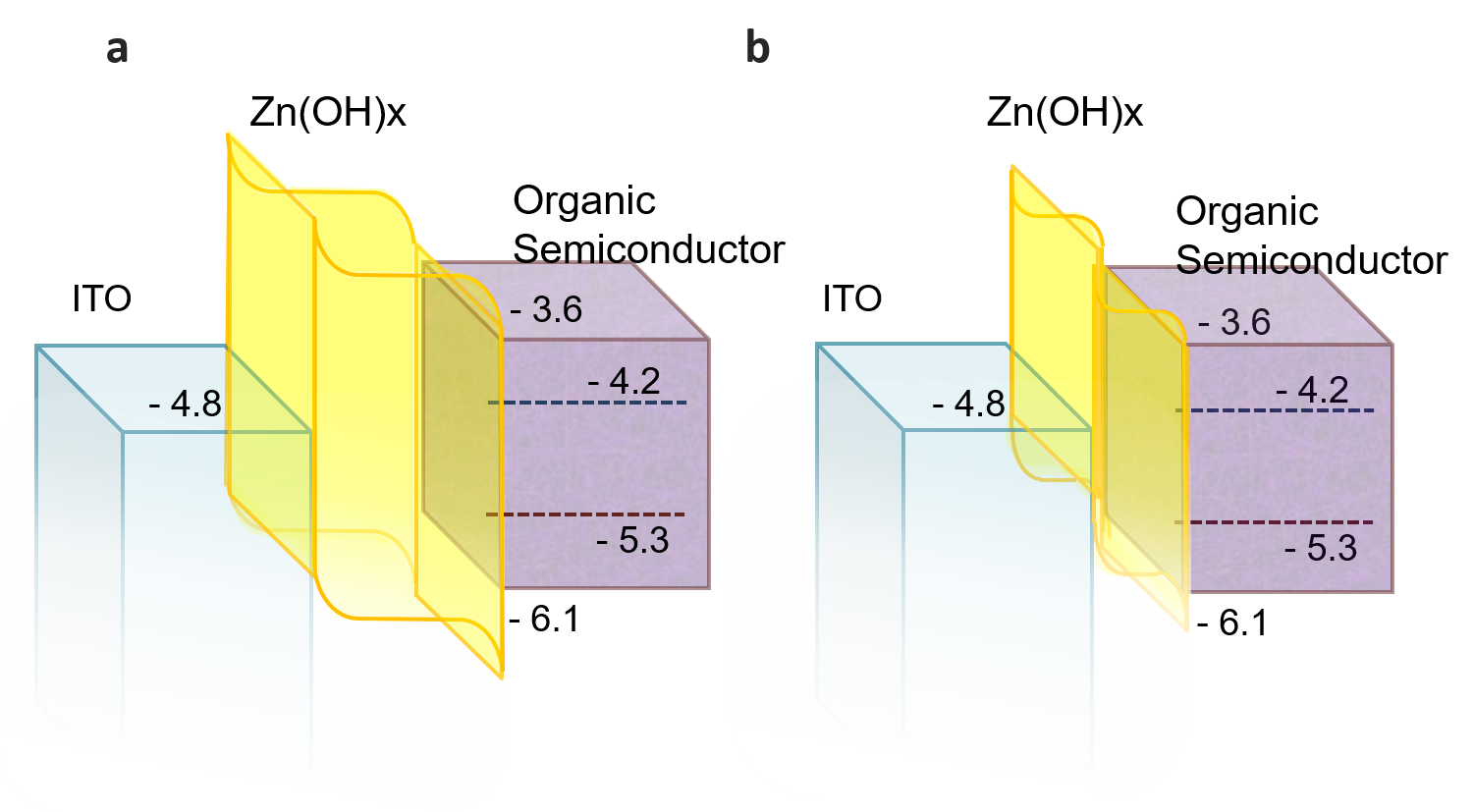


**Figure S2.** Schematic figures of ZnOH_x_ inter layered band structure at the interface with ITO electrode and organic semiconductoers. The band structhre reprisent of (a) thick film and (b) ultra-thin film of inter-layer ZnOH_x_. The band edges at the interface significantly reduce and/or increase with Schottky barrier junction.

The spectra, shown in Figure S3, indicated that the films had high transmittance in the visible light region and absorbed UV light. The calculated optical gaps of the films were over 4.4 eV. Taking into account the fact that calcined ZnO absorbs UV light from 300 nm and below.

**Figure S3.** Transmittance of ZnOH_x_ thin films varied with concentration of the precursor solutions


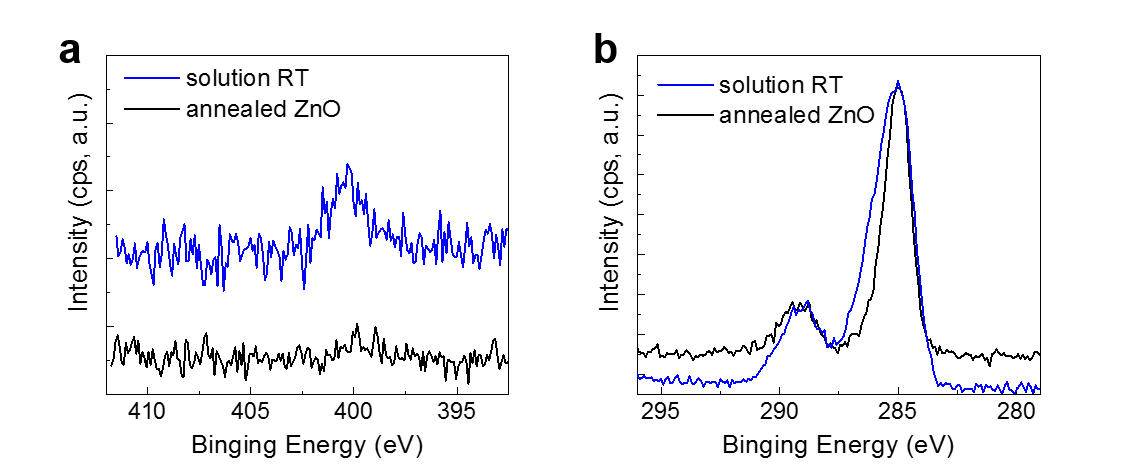
**Figure S4.** XPS analysis of Zn-related thin-films. The local XPS spectra at (a) N 1s and (b) C 1s of ZnOH_x_ thin films and annealed ZnO.


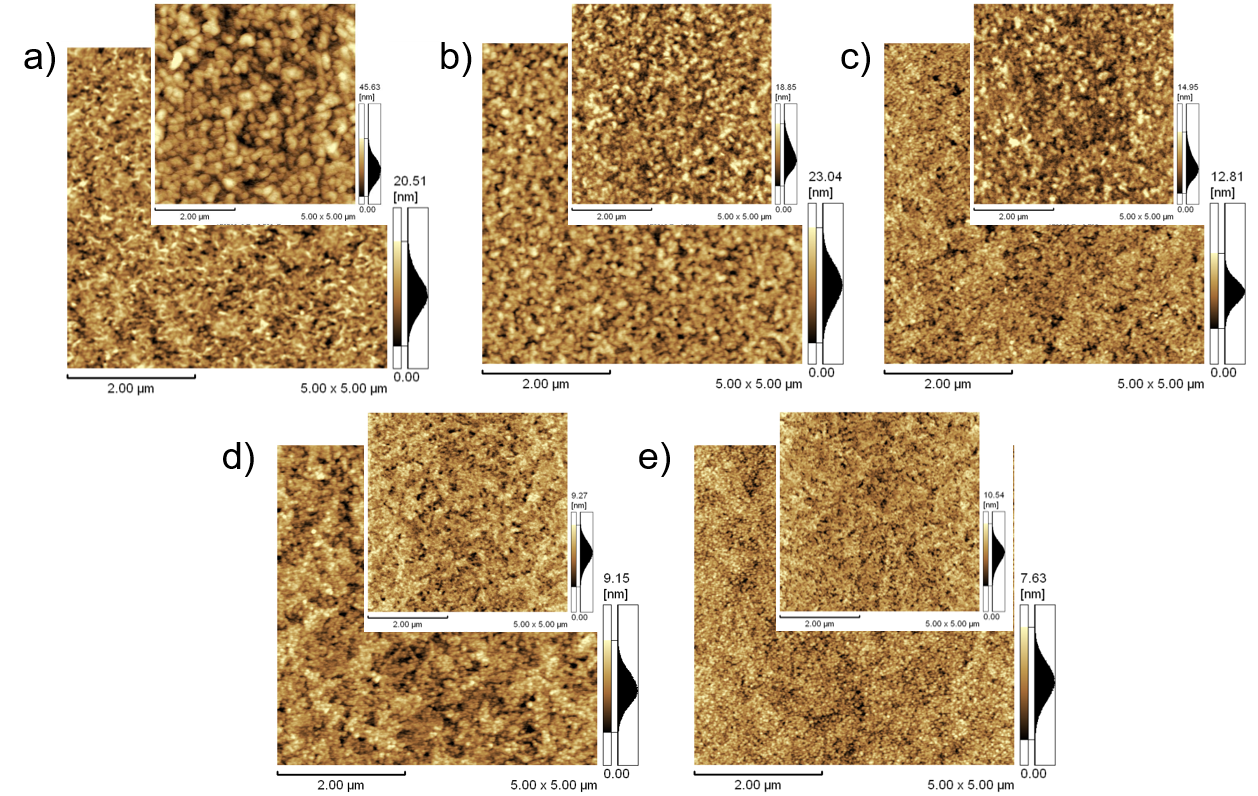


**Figure S5.** Film surface morphology with different concentrations of ZnOH_x_ precursors; a) 100 mM/L, b) 50 mM/L, c) 30 mM/L, d) 20 mM/L and e) 10 mM/L. The insets are surface morphology of ZnOH_x_ film which show poor device performance
